# A new method of recording attendance improves the academic performance of medical students: Student Response

**DOI:** 10.30476/jamp.2020.86659.1259

**Published:** 2021-01

**Authors:** KAMAL DEWAN, NAZIFA BEGUM, INAAM MUNEER MIAN, RAMEEZ SHAHZAD, SAKERIYA FARAH

**Affiliations:** 1 Barts and the London School of Medicine and Dentistry, Garrod Building, Turner Street, Whitechapel, London, E1 2AD, United Kingdom; 2 King’s College London, Guy's Campus, Great Maze Pond, London SE1 1UL, United Kingdom

**Dear Editor**

We appreciate the research conducted by Mondal et al. ( 1 ) which explored how a new attendance system, students’ engagement during the collection of attendance (SEdCA), could be utilised to improve examination performance in medical students based in India. They found that participating in SEdCA improved anatomy test scores in 63 out of 93 students. They investigated how SEdCA could be repurposed outside of taking attendance and we commend the authors for this contribution to medical education. 

We would first like to draw from our experience as senior medical students from London based universities. The issue of attendance is handled via a scanner which scans students’ identification cards. While this is an effective attendance monitoring system, it would not demonstrate the potential academic performance benefit as seen with SEdCA. Although more efficient attendance monitoring is not the centre of discussion here, scanners may be a topic to explore in the future. In light of this issue we recommend a digital quiz. This combats the problem highlighted in the article that teachers invest a large time inspecting roll numbers. One such example of a digital quiz maker is Classmarker ( 2 ), however other brands are available. In our experience, digital quizzes were a common practice during histology lectures which reinforced our learning in that subject.

The quiz could have an access process which requires a student’s roll number and a private code so that only students present in lecture can enter the quiz. The system can also provide an instant feedback list for lecturers to determine who was present. Overall, this speeds up identification of absentees, students answering questions and with the additional benefit of less paper waste compared to SEdCA. 

The article does bring up the concern of cheating. This is possible with the digital questions also. Students who are present could send the private quiz code to absentees giving them access. To remedy this, the code and question can be displayed for a short time. However, cheating will not create a huge issue academically as the purpose of formative assessments is to learn over time rather than attaining high grades as seen with summative examinations.

The article does state that further studies should include other year groups. We find this paramount as first year students are unique compared to other year groups. They are in a transition period and are experiencing medical sschool examinations for the first time. Hill et al. ( 3 ) reinforce this with their study on 987 students. They discovered that the most significant stressor for first year medical students was related to transitioning to medical school. Therefore, differences seen in the test scores could be as a result of increased comfortability with examinations over time. To see the impact of SEdCA, this needs to be controlled for. Our recommendation is to compare the test scores of the same two anatomy tests with the scores from the cohorts above when they were in first year. With this in place, we could observe how students naturally improve over time in their first year and as a result we could more accurately decipher how much impact SEdCA made.

The article states that the tests were based on different anatomy topics. Individual differences will naturally exist in the form of differing strengths and weaknesses within different fields of anatomy. It is possible that some of the observed differences in test scores could be a result of this phenomenon. 

We thank the authors for bringing to light new research into formative assessments and we endorse the idea of having more questions during lectures to help supplement learning over time. 

## References

[1] Mondal H, Saha K, Mondal S, Saha P, Biri S ( 2020). A new method of recording attendance improves the academic performance of medical students. J Adv Med Educ Prof.

[2] Online Quiz Maker Tool [Internet] ClassMarker Online Quiz Maker. 2020 [cited 21 May 2020]. https://www.classmarker.com/online-testing/quiz-maker/.

[3] Hill M, Goicochea S, Merlo L ( 2018). In their own words: stressors facing medical students in the millennial generation. Medical Education Online.

